# Physiological Response of *Populus balsamifera* and *Salix eriocephala* to Salinity and Hydraulic Fracturing Wastewater: Potential for Phytoremediation Applications

**DOI:** 10.3390/ijerph17207641

**Published:** 2020-10-20

**Authors:** Michael A. Bilek, Raju Y. Soolanayakanahally, Robert D. Guy, Shawn D. Mansfield

**Affiliations:** 1 Department of Wood Science, Faculty of Forestry, University of British Columbia, 2424 Main Mall, Vancouver, BC V6T 1Z4, Canada; mabilek@mail.ubc.ca; 2 Indian Head Research Farm, Agriculture and Agri-Food Canada, Indian Head, SK S0G 2K0, Canada; raju.soolanayakanahally@canada.ca; 3 Department of Forest and Conservation Sciences, Faculty of Forestry, University of British Columbia, 2424 Main Mall, Vancouver, BC V6T 1Z4, Canada; rob.guy@ubc.ca

**Keywords:** soil salinity, *Salix* spp., *Populus* spp., biomass production, fracking wastewater, mineral nutrition, gas exchange, metabolite analysis

## Abstract

Natural and anthropogenic soil degradation is resulting in a substantial rise in the extension of saline and industrially-polluted soils. Phytoremediation offers an environmentally and economically advantageous solution to soil contamination. Three growth trials were conducted to assess the stress tolerance of native Canadian genotypes of *Populus balsamifera* L., *Salix eriocephala* Michx., and one hybrid willow (*S. discolor* × *S. dasyclados*) to salinity and hydraulic fracturing (fracking) wastewater. Thirty-three genotypes were grown in NaCl or fracking wastewater solutions between 0 and 7 mS^−1^ over a period of 3–4 months. *P. balsamifera* was observed to be relatively salt-intolerant compared to *S. eriocephala* and hybrid willow, which is likely caused by an inability of *P. balsamifera* to restrict Na^+^ translocation. Photosynthesis and transpiration decreased with salinity treatments, and severe reductions occurred with exposure to fracking solutions. Raffinose and stachyose content was tripled in leaf and root tissues. In willows, Na^+^ was primarily confined to root tissues, Cl^−^ accumulated up to 5% dry weight in leaves, and K^+^ was translocated from roots to leaves. Willow genotypes CAM-2 and STL-2 displayed the greatest maintenance of growth and resistance to necrotic symptoms in all trials, suggesting that these genotypes may be useful for practical application and further field study.

## 1. Introduction

At present, the available arable land is rapidly declining worldwide due, in part, to increased salinity arising from both natural and anthropogenic sources, such as agriculture and oil and gas exploitation activities [1,2,3,4]. Current methods of mechanical and chemical remediation of disturbed soils are prohibitively expensive, laborious, and disruptive to the local ecology. Bioremediation is a less invasive alternative to chemical and mechanical remediation that typically employs the natural processes of microorganisms and fungi to degrade waste [5,6]. Plants can also be used in remediation activities as their macroscopic structures can host microbial communities and may aid in the establishment of successional organisms [7,8,9]. Phytoremediation is the process of improving soil quality using plants via contaminant degradation, stabilization, accumulation, filtration (such as through a constructed wetland), or volatilization. A range of plant species have been identified for potential use in phytoremediation [10,11,12]. However, most native species used for remediation of disturbed sites are susceptible to salt stress, causing declines in germination, establishment, yield, and survival. Salts, in conjunction with pollutants originating from industrial byproducts, cause osmotic stress, cellular toxicity, and nutrient imbalance [13]. Most halophytic species that can withstand these stresses are not appropriate for phytoremediation efforts: they are usually not native to disturbed sites and are often slower growing with lower total biomass accrual. Therefore, it is imperative to identify native plant species with useful traits for phytoremediation.

Members of the *Salicaceae* have shown marked abiotic stress tolerance and have attracted interest for the phytoremediation of saline and industrially polluted land due to a variety of advantageous traits [14,15,16,17]. For example, both poplars and willows exhibit fast and expansive root growth, can propagate via coppicing, have a wealth of genetic resources, and are widespread in the Northern Hemisphere [18]. There exists an expanding body of knowledge regarding salinity tolerance in some poplar and willow species, however, a gap remains for *Populus balsamifera* L. (balsam poplar) and *Salix eriocephala* Michx. (heart leaf willow) which occupy a significant portion of the temperate-boreal forest of North America. By screening for tolerance in native poplar and willow species, there is an opportunity to identify and select genotypes that may be well suited for remediation of industrially disturbed sites. These observations could aid in the selection and breeding of native genotypes for use in amelioration of deteriorated soils, as well as restoration of local biodiversity. Moreover, this information would help elucidate the internal mechanisms that poplar and willow species use to cope with abiotic stress, such as salinity.

Previous studies on balsam poplar examined clinal variation in physiology as well as observed genotypic variation in response to moisture stress [19]. Additionally, balsam poplar has been used as a pioneer species in the reclamation and restoration of logging roads and well pads, providing a foundation for successional species that contribute to remediation [20,21]. These initial observations encourage the investigation of other abiotic stress responses, including potential tolerance to salinity for use in saline well pad and spill site reclamation. The objectives of this study were to observe the physiological response of *Populus balsamifera* and *Salix eriocephala* to salinity and hydraulic fracturing (fracking) wastewater by measuring growth and survival, gas exchange, non-structural carbohydrate production, and nutrient balance. We hypothesized that these genera would display tolerance primarily through the exclusion of salts from aboveground tissues. These results could directly benefit the remediation efforts of the oil and gas industries, contribute to the existing body of knowledge concerning salt tolerance in glycophytic species, and create opportunities for further genetic research on abiotic stress tolerance.

## 2. Materials and Methods 

### 2.1. Cutting Acquisition and Growth Conditions

Poplar and willow genotypes were obtained as dormant cuttings from the Agroforestry Development Centre in Indian Head (Saskatchewan, Canada), where they were grown on a common garden site (Table A1). Cuttings were shipped dormant and then stored in the dark at 4 °C until further use. All experiments were conducted in the University of British Columbia Horticulture Greenhouse (Vancouver, BC, Canada) from 2016–2018.

Dormant cuttings were retrieved from cold storage and a 1 cm fresh cut was made to the bottom of each cutting. 0.4% IBA Stim-Root No. 2 rooting powder (Brampton, Ontario) was applied to the fresh cut before planting into soil with at least three buds exposed. Cuttings were rooted in 8-L pots containing 2:1:1 peat, crushed bark, and pumice. 

All trees were irrigated with 500 mL of half-strength fertilizer solution (Table A2) with or without the addition of salinity treatment per watering event (details below). Natural greenhouse lighting was supplemented by a red/white/blue LED Philips Green lighting system (Markham, ON, Canada) supplying minimum photosynthetically active radiation (PAR) of 300 µmol /m^−2^s^−1^ at the table surface. An 18:6 day/night photoperiod was applied, with an average day/night temperature of 23 °C and 18 °C, respectively.

### 2.2. Experimental Design

Three separate salinity trials were conducted. In the first screening trial, three NaCl salinity treatments (0 [control], 30, and 80 mM) supplemented with half-strength fertilizer were applied to 33 genotypes consisting of four replicates per genotype for eight weeks. Height, diameter, and survival were recorded weekly. For the second salinity trial, seven genotypes were selected from the initial screening trial based on their mortality and height, as well as one hybrid genotype not present in the initial screening trial (Table A1). Ten replicates per genotype were then treated with 0, 20, 40, and 60 mM NaCl solutions supplemented with half-strength fertilizer for 12 weeks. Height, diameter, and survival were again recorded every two weeks, and gas exchange was measured at weeks six and ten. All biomass was harvested at the conclusion of the trial. The final trial consisted of treating the four most promising genotypes with diluted fracking wastewater (FWW, Table A3), whereby 10 replicates per genotype were grown for eight weeks with fracking water supplementation. The FWW was diluted to match the electrical conductivity (EC) of the 20 and 60 mM NaCl treatments in the earlier second trial. In all growth trials, treatment blocks were established randomly, such that each treatment block contained one replicate of every genotype. Within the treatment blocks, individual plants were randomly arranged and treatment delivery was facilitated with dripline irrigation. Height, diameter, and survival were recorded every two weeks, gas exchange was measured at week six, and all biomass was harvested at the conclusion of the trial.

### 2.3. Growth and Biomass Measurements

Plant height was measured from the soil surface to the tip of the most distal leaf of the dominant whip, and diameter was measured with digital calipers at the base of the primary newly-formed shoot. Mortality was determined when necrosis was apparent on approximately >50% of leaf tissue and growth had ceased. All biomass was destructively harvested between 10:00 and 14:00 over nine days. The first three fully developed leaves were removed, weighed, and immediately flash frozen in liquid nitrogen. The stem was cut at the source of the new growth, while the remainder of the leaves were stripped and placed in paper bags with fresh weights of both leaves and stems recorded. Roots were vigorously shaken to remove growth media, washed to remove fine particulate matter, and a small root sample weighed and stored in liquid nitrogen. The remainder of the root tissues were dabbed dry with paper towel, weighed, and stored in paper bags. Dry biomass was recorded after drying leaves, stem and roots in paper bags at 60 °C for several days, once the weight had stabilized.

### 2.4. Gas Exchange Measurements

Gas exchange measurements were taken using a LI-6400XT portable photosynthesis system (LI-COR Biosciences, Lincoln, NE, USA) between the hours of 10:00 and 13:00, with all plants measured in a random order. The first fully developed leaf from the top of the tree was chosen for measurement with the following chamber settings: LEDs delivered 1000 µmol m^−2^s^−1^ PAR, 50–60% relative humidity, 23 °C, 400 ppm CO_2_, with an air flow rate of 500 µmol s^−1^. Net photosynthetic rate (A), transpiration (E), and intercellular CO_2_ were measured, and instantaneous water-use efficiency (WUE) was calculated with the following formula:

WUE = A/E(1)

Gas exchange sample sizes for genotypes at the treatment level varied due to occasional lack of healthy tissue to measure. These unequal samples sizes were accounted for in all statistical analyses.

### 2.5. Elemental Analyses

Dried leaves from individual trees were pooled to ensure homogeneity and finely ground in a Black and Decker^©^ coffee grinder. Dried roots were ground using a Wiley mill to pass a 40-mesh screen and further ground using a 2010 SPEX Sample Prep Geno/Grinder (Metuchen, NJ, USA) at 1500 RPM for 90 s. Samples were analyzed at AGVISE Laboratories (Northwood, ND, USA) using ICP mass-spectrometry to quantify the following elements: N, P, K, S, Ca, Mg, Na, Cl, Zn, Fe, Mn, Cu, B, Pb, and Ni.

### 2.6. Soluble Sugar and Starch Content Quantification

Soluble sugars were quantified using a methanol:chloroform:water (MCW; 12:4:1) extraction and HPLC analyses [22]. Frozen leaf and root tissues were first ground in a Geno/Grinder and kept frozen using liquid nitrogen. Ground tissues were subsequently freeze-dried for 24 h and extracted overnight with 4 mL of MCW. Following extraction, solutions were centrifuged at 6000 RPM and the supernatant was collected. The pellets were then resuspended in 4 mL MCW, centrifuged, and the supernatants collected twice more to yield a total of 12 mL of supernatant. The remaining pellet was set aside and dried at 50 °C overnight for starch determination. Five mL of NanoPure water was added to the supernatant, vortexed, and centrifuged for 6 min at 4000 RPM. The top aqueous layer was then collected and the bottom organic layer was disposed. Two mL of the aqueous layer was vacuum centrifuged overnight and then resuspended in 1 mL of NanoPure water, filtered through a 4.5 µM filter, and 50 µL of 10mg/mL of galactitol was added as an internal standard. Soluble sugars were then quantified using a high-pressure liquid chromatograph (HPLC) fit with a Dionex CarboPac PA1 column and pulsed amperometric detection (Thermo Fischer Scientific, Waltham, MA, USA). HPLC conditions were set to 30 °C, a flow rate of 0.7 mL/min, and an injection volume of 15 µL. The eluent was comprised of 8% 0.2 M sodium hydroxide, 82% degassed NanoPure water, and 10% 20 mM sodium acetate.

Starch content was determined using an acid extraction with fucose as an internal standard [22]. Five mL of 4% sulfuric acid was added to the dried pellet from the MCW extraction and autoclaved at 121 °C for 3 min. After cooling, the solution was centrifuged at 500 RPM, the supernatant was collected and passed through a 4.5 µM filter, and 50 µL of 10 mg/mL stock was added. HPLC detection employed the aforementioned HPLC fit with the Dionex CarboPac PA1 column and guard, with 100% NanoPure water as the mobile phase.

### 2.7. Statistical Analyses

Statistical analyses were performed in R (version 3.3.1). Linear mixed-effect models were developed to test response variables; differences between means were calculated using Type III analysis of variance. *p*-values were adjusted using the Tukey method and tested at a 95% confidence level. Wilks-Shapiro tests were used to test normality. Discrepancies in sample size are explained in Appendix A.

## 3. Results

### 3.1. Growth Performance of Poplar and Willow in Response to Salinity and Fracking Wastewater Treatments

**Figure A1:**
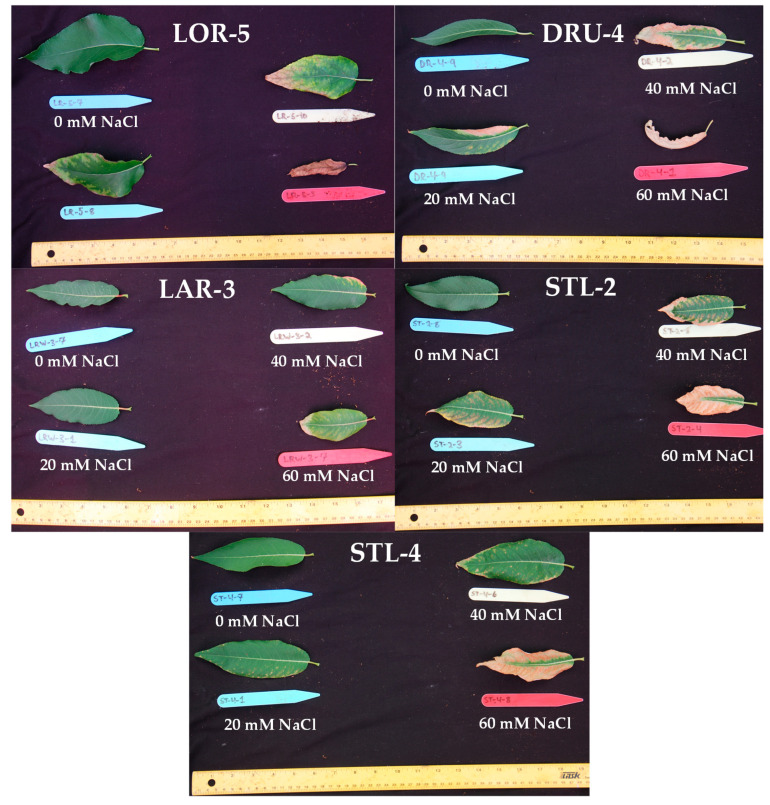
Leaves collected from genotypes LOR-5, DRU-4, LAR-3, STL-2, and STL-4 after 12 weeks of salinity treatment.

In the first salinity trial, clear differences were apparent in the salinity tolerance of balsam poplar compared to willow stecklings, as demonstrated by their growth and survival during eight weeks growth at either 0, 30, and 80 mM NaCl. Balsam poplar experienced complete mortality in the 80 mM NaCl treatments, while the willows suffered only 7% mortality under similar conditions. Furthermore, there was no significant reduction (*p* < 0.05) in the height of willows between the control and 30 mM NaCl treatment, whereas poplar displayed significant height reductions (27.9% and 52.7%) at 30 and 80 mM NaCl treatments, respectively (Table A4). We then decided to perform further salinity trials with a refined selection of genotypes to more closely compare their response to salinity and FWW stresses. Of the poplar genotypes, GPR-10 and LOR-5 were chosen to compare their relatively poor and adequate survival, respectively. CAM-2 and LAR-3 were chosen for their growth characteristics and resistance to necrosis, while DRU-4 was selected for its rapid growth and susceptibility to necrosis, and STL-2 and STL-4 represented a middle point in salinity resistance (Figure A1).

**Figure 1:**
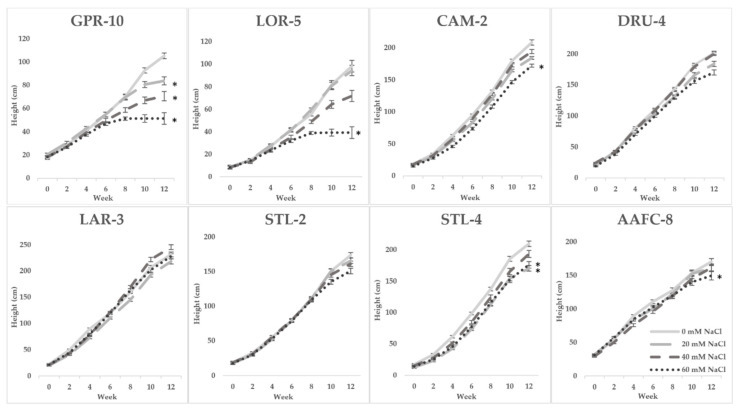
Bi-weekly average height (+/− SEM) of poplar, willow, and hybrid willow genotypes from the second salinity trial. Control, low, moderate, and high treatments represent 0, 20, 40, and 60 mM NaCl, respectively (*n* = 9 for each genotype and treatment). Asterisks denote significance from control treatment at the conclusion of the trial (*p* < 0.05).

**Figure 2:**
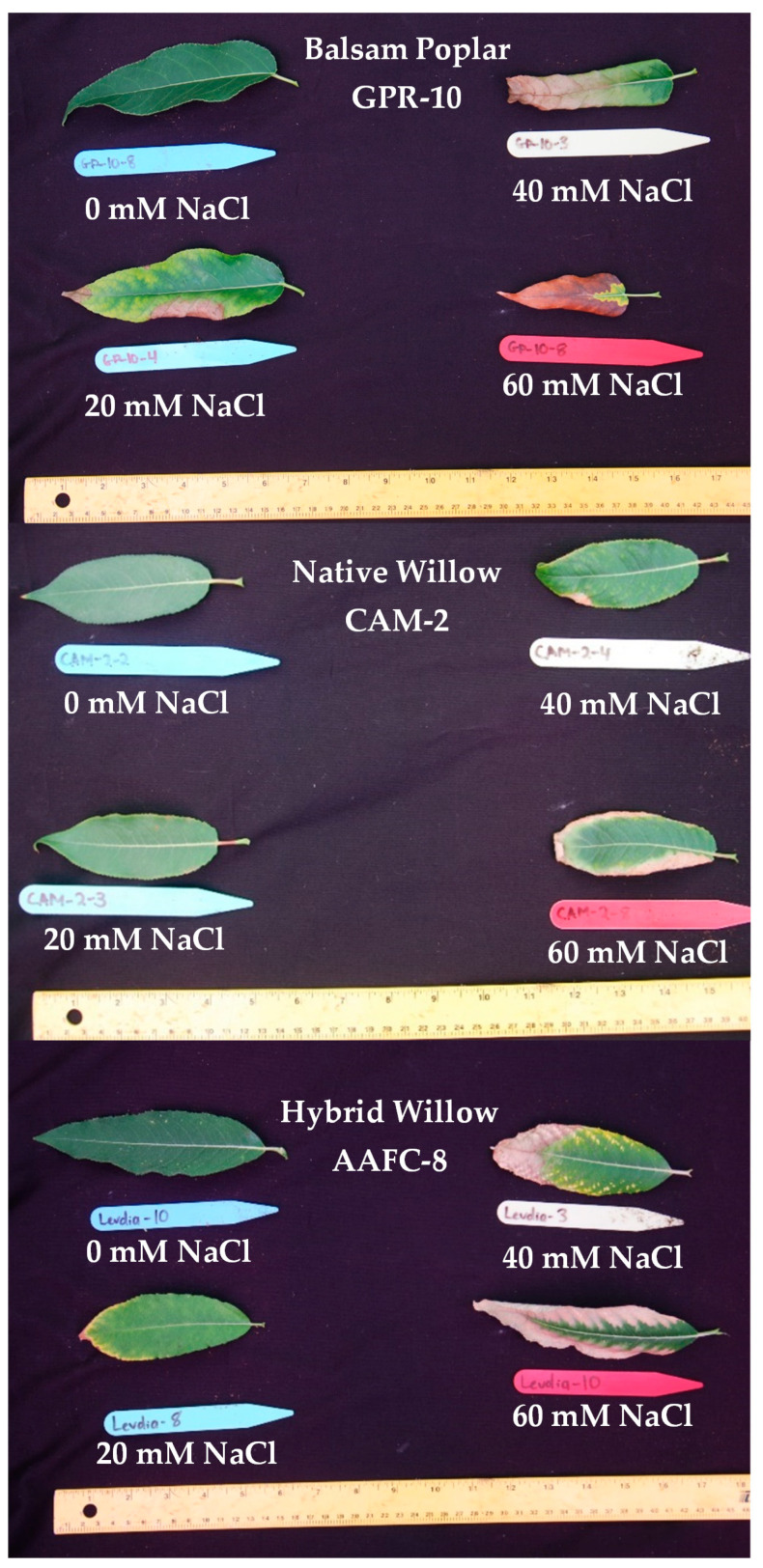
Balsam poplar, native willow, and hybrid willow leaves after 12 weeks of growth with salinity treatments.

Seven of the 32 initially examined genotypes and one hybrid willow were then employed for further treatments at 0, 20, 40, and 60 mM NaCl in a second salinity trial. Fundamentally, growth patterns differed between the two species, but a sharp difference in tolerance was very apparent in their growth when subjected to salt treatment. Native and hybrid willow genotypes experienced no mortality at any level of the salinity treatment, while balsam poplar suffered complete mortality under high salinity treatments. Balsam poplar genotypes GPR-10 and LOR-5 experienced diminished heights of 53% and 60% at 60 mM NaCl compared to control, respectively, while both native and hybrid willows experienced reductions ranging between 12–17% (Figure 1). The hybrid willow (AAFC-8) accumulated greater total dry biomass than all other genotypes and experienced a 53% decrease with 60 mM NaCl treatment compared to control. The primary source of biomass loss with salinity appeared to be largely through leaf necrosis and senescence (Figure 2), of which willow genotypes CAM-2 and STL-2 displayed the greatest resistance (Table 1). There was a 21.4–84.8% reduction in dry root biomass at 40 mM NaCl in all genotypes except AAFC-8, which showed no significant effect (*p* < 0.05).

The four most promising genotypes of the second trial, CAM-2, LAR-3, STL-2, and AAFC-8 were then selected for a trial where diluted fracking wastewater was the source of the salinity for eight weeks. Only the hybrid willow genotype experienced complete mortality from the most severe FWW treatment. STL-2 experienced a significant reduction in height (12.1%; *p* < 0.05) at the lowest FWW concentration. AAFC-8 produced 17.7% more dry biomass under control conditions but was surpassed by the other three willows with FWW treatment. Dry biomass reductions were significant in all genotypes with FWW application, ranging from 58% in STL-2 to 80% in LAR-3 (Table 2). Only CAM-2 maintained root growth at the low FWW treatment compared to control. Both CAM-2 and STL-2 resisted systemic necrosis, while LAR-2 and AAFC-8 proved to be more susceptible (Figure 2 and Figure A1).

### 3.2. Physiological Response to Salinity and Fracking Wastewater Treatments

**Figure 3:**
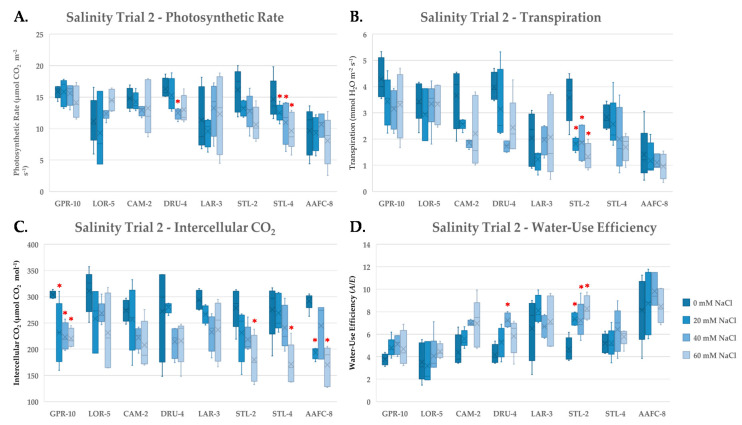
Assimilation rate (**A**), transpiration (**B**), intercellular CO_2_ (**C**), and instantaneous water-use efficiency (**D**) (+/− SEM) of balsam poplar, native, and hybrid willow genotypes recorded at week 6 and of treatment. Control, low, moderate, and high treatments represent 0, 20, 40, and 60 mM NaCl, respectively (n ranged between 4 and 5 for each genotype and treatment). Asterisks represent significant difference from control treatments (*p* < 0.05).

To further examine the stress response of these genotypes to salinity, gas exchange measurements were taken during the second salinity trial at weeks six and ten of the treatment. At week six, a decrease in average net photosynthesis, transpiration, and intercellular CO_2_ was observed, while water-use efficiency (WUE) increased (Figure 3). Net photosynthesis decreased significantly (*p* < 0.05) in willow genotypes DRU-4 and STL-4 with increasing salinity (Figure 3A). While, transpiration decreased most dramatically in willow genotypes CAM-2 and STL-2 (49.5% and 62.8%, respectively, Figure 3B); lowered transpiration resulted in the largest increases in WUE, which were significantly higher (*p* < 0.05) with all treatments in STL-2 compared to control (Figure 3D). Intercellular CO_2_ decreased significantly (*p* < 0.05) at all treatment levels in balsam poplar genotype GPR-10, and at 60 mM NaCl treatments in STL-2, STL-4, and AAFC-8 (Figure 3C). Similar trends were measured at week ten (data not shown), however, average net photosynthesis and transpiration were greater in both control and saline treatments at week six, and no significant treatment effect was apparent at week ten when examining WUE (data not shown).

**Figure 4:**
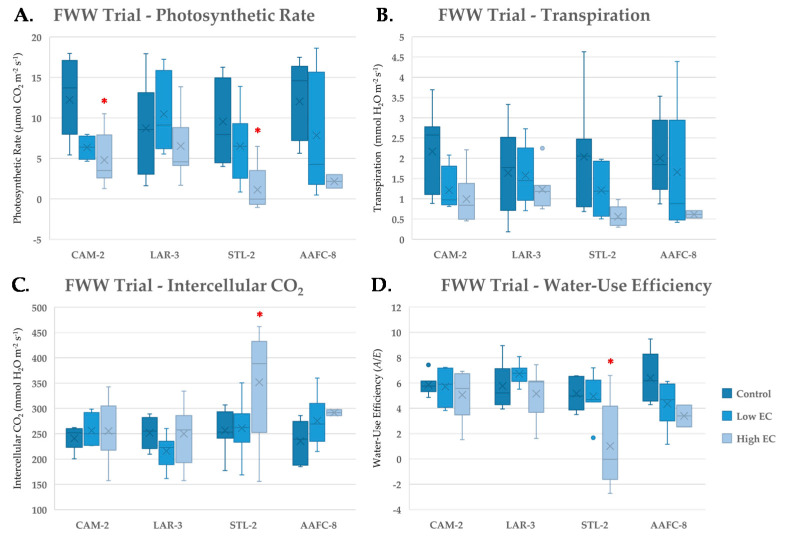
Net photosynthesis (**A**), transpiration (**B**), intercellular CO_2_ (**C**) and instanteous water-use efficiency (**D**) (+/− SEM) of native and hybrid willow genotypes at control, low, and high fracking wastewater treatments following six weeks growth (*n* ranged between 4 and 7 for genotypes and treatments). Mean values for hybrid willow at the high treatment are insignificant due to lack of replicates (*n* = 2). Asterisks denote significant difference from control treatment (*p* < 0.05).

With severe FWW treatment, net photosynthesis significantly decreased by 62.7% and 88.4% (*p* < 0.05) in CAM-2 and STL-2, respectively, but was relatively unchanged in LAR-3 (Figure 4A). Both intercellular CO_2_ and WUE remained unchanged with FWW treatments compared to controls, except in STL-2 (Figure 4C,D), which experienced a significant 70.0% drop (*p* < 0.05) in transpiration (Figure 4B) and 80.8% decrease in WUE at the severe FWW treatment.

### 3.3. Changes in Tissue Nutrient Composition in Response to Salinity and Fracking Wastewater

**Figure 5:**
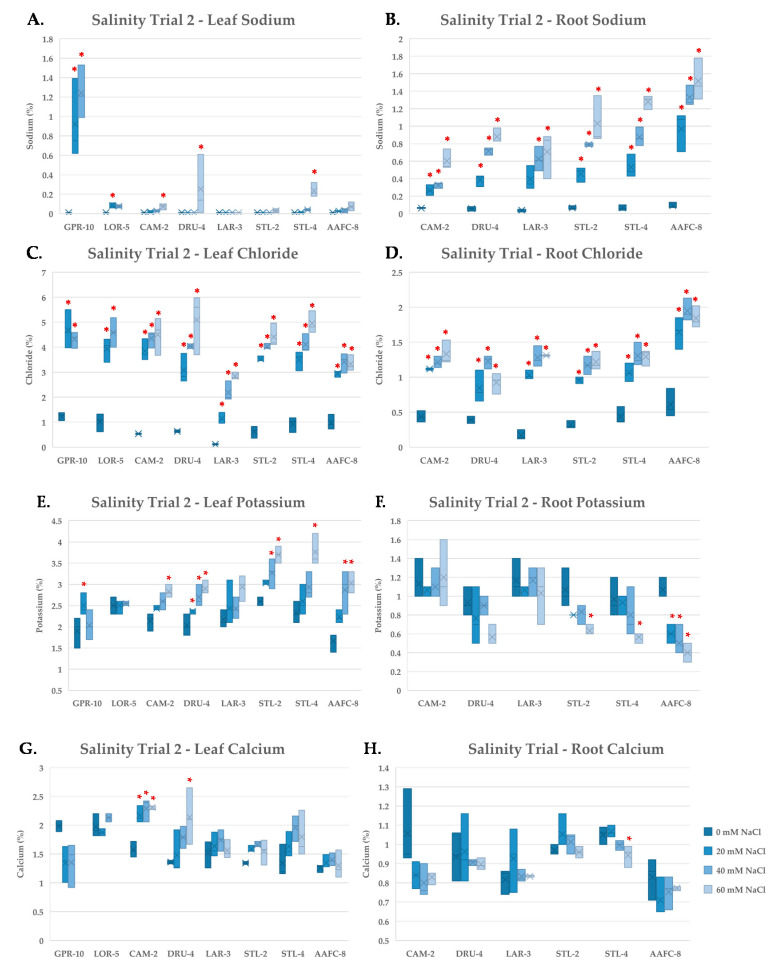
Sodium (**A**,**B**), chloride (**C**,**D**), potassium (**E**,**F**) and calcium (**G**,**H**) content of leaf (**A**,**C**,**E**,**G**) and root (**B**,**D**,**F**,**H**) tissues (+/− SEM) in balsam poplar, native, and hybrid willow genotypes at the conclusion of the second salinity trial. Balsam poplar root tissue is omitted due to lack of tissue resulting from high mortality rates. Control, low, moderate, and severe treatments represent 0, 20, 40, and 60 mM NaCl, respectively (*n* = 3 for each genotype and treatment). Asterisks denote significant difference from control treatment (*p* < 0.05).

Tissue nutrient concentration was examined using ICP-mass spectrometry on independently pooled leaf and root samples in order to gain insights into the physiological response of the balsam poplar and willow genotypes to salinity. Na^+^ did not accumulate in the leaves of the native or hybrid willow genotypes in the second salinity trial when exposed to the 60 mM NaCl treatment (Figure 5A), however, roots did accumulate considerable Na^+^ (Figure 5B). The balsam poplar genotype GPR-10 had over 1% Na^+^ by dry weight in leaf tissues at 40 mM NaCl and also exhibited systemic necrosis of leaf and stem tissues. All genotypes accumulated significantly more (*p* < 0.05) Na^+^ and Cl^−^ in root tissues compared to their controls at all treatment levels (Figure 5B,D).

Chloride reached very high levels in leaf tissues, accounting for up to 5% of the dry weight in DRU-4 and STL-4 in the 60 mM NaCl treatments (Figure 5C). Potassium increased in the leaves of native and hybrid willow genotypes, but not in balsam poplar, and tended to decrease in root tissues except in CAM-2 and LAR-3, which both maintained ~1% K^+^ by dry weight (Figure 5E,F). Calcium concentration was unaffected by salinity treatment in leaves and roots of most genotypes (Figure 5G,H), however, a 52% (*p* < 0.05) increase was observed in the leaves of CAM-2 in the 40 and 60 mM NaCl treatments. No notable changes were apparent in the other nutrients examined.

**Figure 6:**
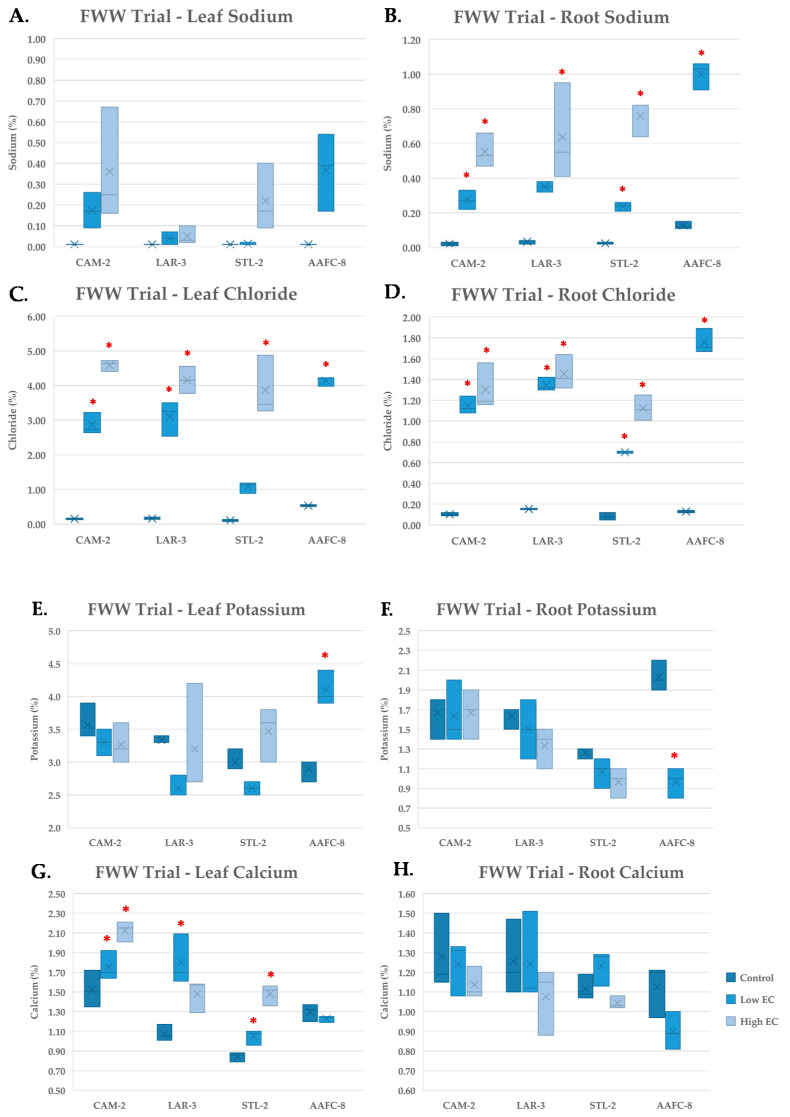
Sodium (**A**,**B**) chloride (**C**,**D**), potassium (**E**,**F**), and calcium (**G**,**H**) content of leaf (**A**,**C**,**E**,**G**) and root (**B**,**D**,**F**,**H**) tissues (+/− SEM) in native and hybrid willow genotypes at the conclusion of the FWW trial treated with control, low, and high fracking wastewater dilutions (*n* = 3 for each genotype and treatment). Hybrid willow leaf and root tissues are omitted due to lack of tissue resulting from high mortality rates. Asterisks denote significant difference from control treatment (*p* < 0.05).

During the FWW treatment, Na^+^ content increased significantly (*p* < 0.05) in the root tissues of all genotypes examined (Figure 6B), but not in leaf tissue (Figure 6A). Chloride concentration reached 4.5% content by dry weight in leaves and 1.5% in roots (Figure 6C,D).

Potassium concentration was largely unchanged in willow genotypes, but AAFC-8 experienced a significant (*p* < 0.05) increase in leaves and decrease in roots at the low FWW treatment (Figure 6E,F). Calcium appeared to be more variable in leaves, increasing significantly (*p* < 0.05) in CAM-2, LAR-3, and STL-2, but not in roots (Figure 6G,H). Copper, lead, and nickel were detected in the stock fracking wastewater, but were not detected at observable quantities in either leaf or root tissues.

### 3.4. Compatible Solute Concentration

In the second salinity trial and FWW trial, starch and six non-structural carbohydrates (myo-inositol, glucose, sucrose, fructose, stachyose, and raffinose) were quantified in both leaf and root tissues. The only observed significant changes in leaf solute content with treatment of second salinity trial were a 357% increase of stachyose in GPR-10 and a 57% decrease of fructose in STL-4 (*p* < 0.05; Table 3). There was a high degree of variability between genotypes, but stachyose levels rose consistently with treatment in willow leaves. In root tissues, myo-inositol, sucrose, and raffinose all accumulated with treatment in willows, and myo-inositol, glucose, and raffinose increased in poplars (Table 4). There were no significant effects on starch concentration in either roots or leaves of all genotypes. 

In response to FWW treatments, a significant decline in glucose was observed in leaf tissues in hybrid willow genotypes under the low FWW treatment (*p* < 0.05, Table 5). Myo-inositol and sucrose both increased in roots with severe FWW treatment, but no other trends were observed in the other compatible solutes quantified, or in starch (Table 6).

## 4. Discussion

The salt tolerance of *Populus balsamifera* is not well reported, nor is the effect of fracking wastewater on terrestrial plant species [23]. Furthermore, an examination of the salinity tolerance of native Canadian *Salix eriocephala* genotypes contributes to a limited body of knowledge while providing solutions for industrial reclamation [24]. Salinity treatments ranging from 20 to 80 mM NaCl were chosen to parallel salinity studies performed on closely related species [25,26,27]. We found that the salt treated willows demonstrated a far greater tolerance to saline conditions than the balsam poplar, and that balsam poplar is likely unsuitable for reclamation of Na^+^ dominated soils.

Survival is a crucial parameter in assessing the potential of a species for soil remediation. In this study, balsam poplar genotypes experienced 0% survival at or above 60 mM NaCl indicating that it is a relatively salt-intolerant species, while the comparatively salt-resistant willow genotypes displayed 93% survival following eight weeks treatment with 80 mM NaCl (7.0 mS^−1^). Interestingly, a recent study using an eastern population of *Salix eriocephala* reported a 40% survival rate after 25 days of 3 mS^−1^ saline treatment [24]. Differences between two populations suggest substantial genotypic variance, possibly arising from differing selective pressures between eastern and western Canadian genotypes, such as the prevalence of saline soils and drier climates characteristic of the Canadian prairies [28].

The toxic effects of salinity on plants manifest primarily in stunting or cessation of growth, and decreased carbon assimilation [29,30]. In the second salinity trial, hybrid willow accumulated more biomass at all salinity treatments compared to other genotypes, accruing ~20% greater dry biomass in the control treatment than the next largest genotype (Table 1). Additionally, AAFC-8 experienced no significant reduction in root biomass at either 20 or 40 mM NaCl compared to the corresponding control. High growth rates are advantageous for phytoremediation as absorbed salts can be diluted within plant tissues, mitigating toxic effects as well as providing a secondary product for industry [31,32]. Willow genotypes STL-2 and CAM-2 exhibited average dry biomass losses of only 32.6% and 39.9% at 60 mM NaCl, substantially lower than the 80% decreases observed in LAR-3 and DRU-4 when subject to similar treatments.

One of the largest factors resulting in losses in biomass is salt-induced necrosis and abscission of leaves, which physically releases biomass and limits carbon uptake [33]. We observed salinity-induced senescence followed by the emergence of new leaves in the willow genotypes consistent with previous observations [34]. This phenomenon likely contributed to the overall salt tolerance by shedding Na^+^ laden leaves and replacing them with new, unaffected foliage, thus maintaining carbon assimilation.

Photosynthesis is particularly susceptible to saline soils due to both toxic and osmotic effects. We observed a decrease in net photosynthesis with salinity treatment, consistent with previous findings [32,35,36]. Stomatal closure is triggered via the ABA signalling pathway in the presence of saline soils in order to preserve water in unfavourable osmotic conditions [37]. Wide variation in transpiration rate was observed with salinity treatment, ranging from a 2.9% decrease in GPR-10 to a 62.7% reduction in STL-2, the latter indicating considerable stomatal closure. We examined both transpiration and intercellular CO_2_ concentrations, which can provide insight into the primary cause of the observed decrease in carbon assimilation [38]. Intercellular CO_2_ decreased in all genotypes with increasing salinity, indicating that reductions in stomatal conductance were more impactful than direct effects on photosynthetic capacity. Reduced transpiration, however, may be beneficial in concurrently limiting salt translocation to sensitive tissues [39]. Willow genotypes CAM-2 and STL-2 both exhibited the strictest stomatal control and also exhibited the smallest decreases in biomass with salinity, suggesting that these two genotypes excelled at limiting water loss in saline conditions, and also restricted salts from accumulating in toxic quantities within their photosynthetic tissues.

Sodium ions exert toxic effects through their competition with K^+^ binding sites, degradation of membranes, evolution of reactive oxygen species, and inhibition of photosynthesis [3,40,41,42]. In the native and hybrid willow genotypes, Na^+^ was limited in leaf tissues and accumulated primarily in root tissues. Balsam poplar genotype GPR-10 failed to restrict Na^+^ movement to leaves at 20 mM NaCl and was also the most salt-sensitive genotype examined, however, native willow genotypes DRU-4 and STL-4 amassed Na^+^ in their leaves at the severe salinity treatment and experienced significant biomass loss and senescence, suggesting that that control of Na^+^ translocation is crucial for salt tolerance within these species.

Sodium and potassium are highly competitive for binding sites on membrane-bound proteins and cytosolic enzymes, and plants have evolved highly specialized systems to maintain a high K^+^:Na^+^ ratio [43]. We observed an increase in K^+^ concentration in leaves with increasing salinity in willow genotypes, but not in balsam poplar. Roots experienced a decline in K^+^ with salinity, suggesting translocation to leaf tissues to help maintain metabolic function. A favourable K^+^:Na^+^ ratio was apparent in willow leaves, and likely contributes to normal enzyme function, preventing apoptosis and necrotic lesions from forming. Calcium was seen to increase in willow leaves, significantly at all salinity levels in the salt-tolerant CAM-2 genotype. Calcium acts as a secondary messenger in the salt stress response and contributes to membrane stabilization [44,45].

Chloride accumulated to levels as high as 5% by dry weight in leaves, and to far lower concentrations in roots. It has been demonstrated that Cl^−^ facilitates stomatal closure [46]. Furthermore, it may act as a readily available ion used in internal osmotic adjustment for maintenance of turgor and facilitating water uptake. Chloride is toxic in tolerant species at tissues concentrations of 15–50 mg/g of dry weight, and was likely exerting toxic effects on the salt-treated trees [47]. 

Plants can produce internal osmotic adjustments via synthesis of a wide variety of compatible solutes including sugars, sugar alcohols, and charged metabolites [48]. We measured the concentrations of soluble carbohydrates in response to increasing salinity and found an increase of stachyose in leaf tissues and myo-inositol and raffinose in roots. Both stachyose and raffinose have been shown to act as osmoprotectants and free-radical scavengers, which may aid in anti-oxidant defense as well as promote normal enzyme functions [49,50]. It should be noted that the 357% increase in stachyose content may be misleading, as the concentrations measured were under 5mg per gram of tissue. Sucrose content was elevated in the roots of native willow genotypes, but not in balsam poplar, and may have contributed to tolerance via mild osmotic adjustments, via the production of stachyose and raffinose, and metabolization to yield energy [51]. Despite the variety of metabolites examined in this study, little change was observed, suggesting that these metabolites likely contribute only marginally to overall salinity tolerance.

Four genotypes displayed a variety of traits that could be advantageous for industrial reclamation and phytoremediation: CAM-2, LAR-3, STL-2, and AAFC-8. Fracking wastewater is a complex mixture of highly concentrated salts, as well as surfactants, lubricants, and biocides, and the composition changes with industrial site and process [52]. Subjecting these four tolerant genotypes to FWW treatments allowed us to not only test their growth on an industrial pollutant, but also observe and compare their physiological stress response to that of saline water.

Numerous differences were witnessed with FWW compared to salinity treatments. Despite good performance in the first two salinity trials, the hybrid willow genotype failed to survive after eight weeks of fracking wastewater treatment. This genotype experienced a 44.2% loss of total dry biomass at the low FWW treatment compared to a 25.8% decrease at salinity treatments with similar EC. Although the ECs of the severe FWW treatment and 60 mM NaCl solutions were similar, the actual Na^+^ content of the severe FWW treatment was calculated to be 72.8 mM, which may be too toxic for the hybrid willow to withstand, or the other toxic constituents in the FWW may have contributed to its mortality. However, CAM-2, LAR-3, and STL-2 survived eight weeks of the severe FWW treatment and mirrored the growth of earlier salinity trials. Both CAM-2 and STL-2 exhibited a more pronounced reduction in biomass compared to the previous salinity trials, but also displayed fewer necrotic lesions. This suggests that energy that may normally be used for growth could instead be allocated to active transport processes and antioxidant defenses [53].

Net photosynthetic rates were heavily reduced in all genotypes with FWW treatment, except in LAR-3. Negative photosynthetic rates were recorded in STL-2 indicating that respiration outpaced carbon assimilation, and supports the idea that energy is being allocated to active stress responses, or that additional stressors are interfering with metabolic processes [54]. Transpiration paralleled the previous salinity trials, but WUE increased and intercellular CO_2_ was unchanged between FWW treatments. This indicates that there was no significant stomatal restriction to CO_2_, rather the stressors in the FWW directly impacted the photosynthetic capacity [53,55,56].

Sodium and chloride both followed analogous patterns of accumulation in leaf and root tissues treated with FWW compared with the previous salinity trial. The hybrid willow accumulated more leaf Na^+^ at the low FWW treatment than it had under the previous 60 mM NaCl treatment, indicating that the additional stressors present in the FWW may be disrupting normal Na^+^ compartmentalization. Potassium remained unchanged in response to FWW treatments but was present in higher concentrations than in the salinity trials, likely due its presence in the FWW in addition to the applied fertilizer. Calcium also remained unchanged despite its greater abundance in the FWW solution, suggesting that it may exist in a biologically unavailable form. Copper, lead, and nickel were found to be present in low concentrations in the stock FWW solution, but were not found in detectable quantities in the plant tissues examined.

A growing body of work has documented the production of antioxidants, phytochelators, and various charged metabolites in response to salinity stresses, but non-structural carbohydrates have been poorly studied. Roots accumulated sucrose in response to FWW treatment which may be used to fuel respiration and provide ATP for active transport and production of other osmolytes [51,54]. Fructose and raffinose both decreased in leaf tissues with FWW treatment, but were unaffected in the salinity trials; similar decreases were found in cucumbers grown near an industrial copper production site [57]. The willow hybrid exhibited a 50% decrease in myo-inositol and glucose as well as an 80% drop in raffinose with the low FWW treatment, while CAM-2 and STL-2 maintained greater carbohydrate concentrations at the severe FWW treatment.

## 5. Conclusions

This research identified native genotypes that may ultimately be used in ecological restoration of disturbed and marginal landscapes in the northern boreal forest and plains. Genotypes CAM-2 and STL-2 exhibited the greatest tolerance to both salinity and FWW stresses, displaying advantageous traits for phytoremediation such as maintenance of growth and biomass, high rates of Na^+^ and Cl^−^ accumulation, and resistance to tissue necrosis and senescence. Further research is necessary to elucidate the specific mechanisms of salinity tolerance in these individual genotypes, however, this study illustrates the adaptive strategies that poplar and willows use in salt tolerance, and provides resources that could be used in breeding programs for selection of trees for otherwise unproductive sites.

## Figures and Tables

**Table 1:** Average total dry biomass (A), leaf (B), stem (C), and root biomass (D) of poplar, willow, and hybrid willow genotypes from the second salinity trial (*n* = 9 for each genotype and treatment).

A.		Total Dry Biomass (g)				B.		Dry Leaf Biomass (g)			
Species	Genotype	0 mM NaCl	20 mM NaCl	40 mM NaCl	60 mM NaCl	Species	Genotype	0 mM NaCl	20 mM NaCl	40 mM NaCl	60 mM NaCl
Balsam poplar	GPR-10	47.1 ^a^	16.4 ^b^	9.9 ^bc^	2.3 ^c^	Balsam poplar	GPR-10	25.8 ^a^	9.0 ^b^	5.6 ^bc^	1.1 ^c^
	LOR-5	23.8 ^a^	15.9 ^b^	8.3 ^bc^	0.9 ^c^		LOR-5	12.8 ^a^	8.6 ^b^	3.3 ^c^	0.3 ^c^
Native willow	CAM-2	64.1 ^a^	45.9 ^ab^	49.5 ^b^	38.5 ^b^	Native willow	CAM-2	21.8 ^a^	14.8 ^ab^	17.2 ^b^	14.2 ^b^
	DRU-4	61.9 ^a^	49.8 ^a^	36.9 ^b^	15.8 ^c^		DRU-4	20.3 ^a^	17.9 ^a^	13.2 ^b^	5.4 ^c^
	LAR-3	71.2 ^a^	54.7 ^ab^	48.5 ^bc^	31.8 ^c^		LAR-3	18.2 ^a^	14.1 ^a^	12.4 ^ab^	7.3 ^b^
	STL-2	46.5 ^a^	40.1 ^ab^	39.4 ^ab^	31.3 ^b^		STL-2	15.7 ^a^	14.8 ^a^	14.7 ^a^	12.8 ^a^
	STL-4	72.7 ^a^	44.5 ^b^	43.2 ^bc^	28.7 ^c^		STL-4	24.1 ^a^	15.5 ^b^	13.4 ^b^	9.2 ^c^
Hybrid willow	AAFC-8	90.6 ^a^	67.2 ^b^	53.7 ^bc^	42.6 ^c^	Hybrid willow	AAFC-8	35.7 ^a^	23.9 ^b^	20.3 ^b^	18.7 ^b^
**C.**		**Dry Stem Biomass (g)**				**D.**		**Dry Root Biomass (g)**			
Species	Genotype	0 mM NaCl	20 mM NaCl	40 mM NaCl	60 mM NaCl	Species	Genotype	0 mM NaCl	20 mM NaCl	40 mM NaCl	60 mM NaCl
Balsam poplar	GPR-10	10.6 ^a^	3.6 ^b^	2.5 ^b^	1.1 ^b^	Balsam poplar	GPR-10	4.6 ^a^	0.7 ^b^	0.5 ^b^	
	LOR-5	6.7 ^a^	5.5 ^ab^	3.0 ^ab^	0.4 ^b^		LOR-5	2.1 ^a^	0.7 ^b^	0.3 ^b^	
Native willow	CAM-2	26.3 ^a^	20.0 ^ab^	21.4 ^bc^	14.7 ^c^	Native willow	CAM-2	8.4 ^a^	6.6 ^ab^	6.5 ^ab^	4.7 ^b^
	DRU-4	21.2 ^a^	17.4 ^ab^	15.5 ^b^	6.2 ^c^		DRU-4	8.7 ^a^	5.1 ^b^	3.3 ^bc^	1.3 ^c^
	LAR-3	28.6 ^a^	24.6 ^ab^	29.1 ^a^	18.2 ^b^		LAR-3	12.4 ^a^	8.1 ^b^	6.2 ^bc^	3.7 ^c^
	STL-2	19.0 ^a^	15.0 ^ab^	14.4 ^ab^	11.6 ^b^		STL-2	7.6 ^a^	4.9 ^b^	4.3 ^b^	2.6 ^b^
	STL-4	27.8 ^a^	16.2 ^b^	19.6 ^b^	13.7 ^b^		STL-4	10.8 ^a^	5.5 ^b^	4.6 ^bc^	2.4 ^c^
Hybrid willow	AAFC-8	29.7 ^a^	23.7 ^ab^	20.7 ^b^	12.8 ^c^	Hybrid willow	AAFC-8	11.3 ^a^	11.1 ^a^	8.6 ^a^	4.1 ^b^

Mean values sharing a superscript letter in the same row are not significantly different (*p* < 0.05).

**Table 2:** Average total dry biomass (A), leaf (B), stem (C), and root (D) biomass of native and hybrid willow genotypes in the control, low and high fracking wastewater treatments (*n* = 5; CAM-2 and STL-2, and *n* = 8; LAR-3 and AAFC-8). Superscripts denote significance from corresponding control treatment (*p* < 0.05).

A.	Total Dry Biomass (g)				B.	Dry Leaf Biomass (g)			
Species	Genotype	Control	Low EC	High EC	Species	Genotype	Control	Low EC	High EC
Native willow	CAM-2	36.7 ^a^	26.5 ^b^	11.3 ^c^	Native Willow	CAM-2	18.0 ^a^	12.4 ^b^	5.5 ^c^
	LAR-3	40.2 ^a^	28.2 ^b^	7.9 ^c^		LAR-3	15.7 ^a^	10.3 ^b^	2.6 ^c^
	STL-2	39.6 ^a^	25.5 ^b^	16.5 ^c^		STL-2	17.6 ^a^	11.9 ^b^	8.8 ^b^
Hybrid willow	AAFC-8	47.3 ^a^	26.4 ^b^		Hybrid willow	AAFC-8	24.9 ^a^	11.4 ^b^	
**C.**	**Dry Stem Biomass (g)**				**D.**	**Dry Root Biomass (g)**			
Species	Genotype	Control	Low EC	High EC	Species	Genotype	Control	Low EC	High EC
Native willow	CAM-2	16.0 ^a^	11.6 ^a^	4.8 ^b^	Native willow	CAM-2	2.7 ^a^	2.5 ^a^	1.3 ^b^
	LAR-3	20.2 ^a^	14.9 ^b^	5.5 ^c^		LAR-3	4.3 ^a^	3.1 ^b^	0.9 ^c^
	STL-2	18.2 ^a^	10.8 ^b^	6.3 ^c^		STL-2	3.8 ^a^	2.8 ^b^	1.8 ^c^
Hybrid willow	AAFC-8	18.4 ^a^	12.0 ^b^		Hybrid willow	AAFC-8	4.0 ^a^	3.0 ^b^	

Mean values sharing a superscript letter in the same row are not significantly different (*p* < 0.05).

**Table 3:** Soluble sugar and starch contents in poplar, willow, hybrid willow leaf tissues at the conclusion of the second salinity trial. Willow and hybrid leaves were analyzed at 0 and 60 mM NaCl, and poplar leaves at 0 and 40 mM NaCl due to lack of available leaf tissue (*n* = 3 for each genotype and treatment). Asterisks denote significant difference from control treatment (*p* < 0.05). One sample outlier was removed for DRU-4 glucose in the high fracking wastewater treatment.

			Salinity Trial 2—Leaf Tissue					
Treatment	Genotype	Myo-Inositol (mg/g)	Glucose (mg/g)	Sucrose (mg/g)	Fructose (mg/g)	Raffinose (mg/g)	Stachyose (mg/g)	Starch (%)
0 mM NaCl	GPR-10	7.8	13.4	49.9	6.6	1.4	0.7	1.6
	LOR-5	11.6	30.6	83.6	9.5	0.6	1.3	1.2
	CAM-2	13.0	8.1	78.1	10.1	2.2	1.2	6.3
	DRU-4	9.5	3.0	70.7	2.7	1.7	1.0	4.5
	LAR-3	11.3	10.3	61.7	12.2	1.0	0.5	6.0
	STL-2	9.4	6.9	58.5	7.0	1.4	0.4	4.6
	STL-4	9.4	6.6	79.9	7.7	3.6	0.6	7.6
	AAFC-8	13.2	11.8	64.1	10.6	0.7	2.0	9.0
40 mM NaCl	GPR-10	12.7	11.4	67.6	7.9	1.1	3.2 *	2.2
	LOR-5	15.5	30.9	107.3	14.2	1.3	6.6	1.0
60 mM NaCl	CAM-2	8.7	7.0	61.5	8.1	1.2	3.6	7.4
	DRU-4	12.3	9.9	65.7	10.1	1.0	3.0	6.7
	LAR-3	11.7	14.7	61.2	17.6	0.9	2.5	6.7
	STL-2	7.9	4.4	43.7	3.9	0.9	1.5	5.1
	STL-4	6.8	4.1	39.9	3.3 *	1.0	1.5	10.3
	AAFC-8	9.0	10.4	53.7	9.0	0.8	1.1	8.6

**Table 4:** Soluble sugar and starch contents in poplar, willow, hybrid willow root tissues at the conclusion of the second salinity trial. Willow and hybrid roots were analyzed at 0 and 60 mM NaCl, and poplar roots at 0 and 40 mM NaCl due to limitations of available root tissue (*n* = 3 for each genotype and treatment). Asterisks denote significant difference from control treatment (*p* < 0.05).

			Salinity Trial 2—Root Tissue					
Treatment	Genotype	Myo-Inositol (mg/g)	Glucose (mg/g)	Sucrose (mg/g)	Fructose (mg/g)	Raffinose (mg/g)	Stachyose (mg/g)	Starch (%)
0 mM NaCl	GPR-10	1.2	4.3	23.1	2.9	0.8	0.1	1.3
	LOR-5	0.8	6.1	21.1	6.9	0.4	0.1	1.4
	CAM-2	0.7	2.9	15	2.5	0.4	<0.1	3.6
	DRU-4	0.6	2.4	22.8	2.4	0.6	0.4	3.5
	LAR-3	0.3	2.9	16.4	3.2	0.3	<0.05	2.2
	STL-2	0.8	2.1	19.6	1.9	0.7	0.1	4.2
	STL-4	1.3	3.2	19.8	4	0.7	0.1	1.7
	AAFC-8	2	3.7	27.6	4.2	0.8	0.1	3.7
40 mM NaCl	GPR-10	3.1	8	22.4	3.7	0.5	0.2	1.4
	LOR-5	1.7 *	8.2	33.2	2.9	1.4	0.6	2.1
60 mM NaCl	CAM-2	3.2 *	3.1	31.7 *	3.1	1.2 *	0.1	6.1
	DRU-4	1.5	3.1	25.1	2	1.6	0.1	1.1
	LAR-3	2.5	3.4	35.3	3.9	2.4 *	0.3 *	2.1
	STL-2	2.9	3.1	34.6 *	3.5	1.7	0.2	2.1
	STL-4	1.9	2.4	24.1	2.5	1	<0.05	1.6
	AAFC-8	2.5	2.6	23.5	2.5	0.7	<0.05	1.5

**Table 5:** Soluble sugar content of willow and hybrid willow leaf tissues at the conclusion of the FWW trial treated with control, low, and high fracking wastewater treatments (*n* = 3 for genotypes and treatments). Asterisks indicate significant treatment effect compared to control (*p* < 0.05). AAFC-8 is omitted at the high fracking wastewater treatment due to limitations in available tissue.

			FWW Trial—Leaf Tissue					
Treatment	Genotype	Myo-Inositol (mg/g)	Glucose (mg/g)	Sucrose (mg/g)	Fructose (mg/g)	Raffinose (mg/g)	Stachyose (mg/g)	Starch (%)
Control	CAM-2	10.5	23.2	64.3	19	4.2	0.6	2.4
	LAR-3	14.1	25.1	91.8	21.5	5.3	0.6	2
	STL-2	13.8	18.1	100.3	16.2	4.8	1.5	2.6
	AAFC-8	15.1	17.8	92.5	14.5	4.8	1.3	4.9
Low EC	CAM-2	9.5	10.6	84.0	11.7	1.8	0.8	2.3
	LAR-3	15.2	23.1	96.7	18.9	3.8	1.5	4.3
	STL-2	11.0	16.6	71.0	14.6	1.9	1.0	2.2
	AAFC-8	7.3	10.8 *	97.3	8.5	0.9	1.0	2.7
High EC	CAM-2	11.1	12.8	95.6	11.9	1.1	0.9	3.6
	LAR-3	11.4	9.9	80.3	8.2	0.6	0.7	3.1
	STL-2	15.6	14.7	111	11.1	1.8	1.2	3.6

**Table 6:** Soluble sugar content of willow and hybrid willow leaf tissues at the conclusion of the FWW trial treated with control, low, and high fracking wastewater treatments (*n* = 3 for genotypes and treatments). Asterisks indicate significant treatment effect compared to control (*p* < 0.05). AAFC-8 is omitted at the high fracking concentration due to limitations in available tissue.

			FWW Trial—Root Tissue					
Treatment	Genotype	Myo-Inositol (mg/g)	Glucose (mg/g)	Sucrose (mg/g)	Fructose (mg/g)	Raffinose (mg/g)	Stachyose (mg/g)	Starch (%)
Control	CAM-2	2.3	6.4	9.8	3.4	1	0.3	2.1
	LAR-3	1.6	4.6	14	3.9	1.6	0.4	1.9
	STL-2	2.9	5.7	18	2.5	2.9	1	1.9
	AAFC-8	2.6	5.8	17.1	4.7	1.1	0.5	2.1
Low EC	CAM-2	3.5	5.3	40.1	2.6	1.6	0.4	2.6
	LAR-3	2.6	5.3	25.1	3.7	1.9	0.4	2.5
	STL-2	2.6	6.7	20	4.5	1.6	0.5	1.6
	AAFC-8	4.2	7.4	20.5	4.2	0.6	1.3	2.7
High EC	CAM-2	5	5.7	46.3 *	4.8	1.3	0.2	2
	LAR-3	2.7	5.2	17.6	2.5	1.6	0.1	2.5
	STL-2	3.8	9.3	34	5	0.7 *	0.1	1.6

## Appendix A

Note on sample sizes: six weeks into the second salinity trial, one random replicate of each genotype at all treatment levels was destructively sampled for an experiment not associated with this study, hence the discrepancy between the initial 10 replicates grown and 9 reported replicates in Table 1. Due to the harmful effect of FWW on plant health, insufficient replicates were available for gas exchange measurements on hybrid willow genotype AAFC-8, which impacted our statistical calculations. A simultaneous, collaborative study necessitated the destructive harvest of trees in the FWW trial. Two random replicates of each genotype, at each treatment level, were destructively sampled at week 6 of treatment, and three more random replicates were destructively sampled from CAM-2 and STL-2 prior to the conclusion of the trial.

**Table A1:** Balsam poplar, native and hybrid willow genotypes used in salinity trials. Superscripts indicate genotypes that were grown in experiments 2 and 3, no superscript indicates use in only the first screening trial.

Balsam Poplar	Native Willow	Hybrid Willow
Boyle (BOY)—6	Camrose—(CAM) 2^(2,3)^, 5, 7	LEV-D5 (*S. discolor*) × India(*S. dasyclados*)—13^(2,3)^ (AAFC-8)
Fort McMurray (FTM)—1, 2, 6	Drumheller (DRU)—2, 4^(2)^, 6	
Fort Nelson (FNO)—1, 5, 7	La Ronge (LAR)—1, 3^(2,3)^, 5	
Grand Prairie (GPR)—2, 10^(2)^	Prince Albert (PAL)—1, 4	
La Ronge (LOR)—3, 5^(2)^	Stettler (STL)—2^(2,3)^, 3, 4^(2)^	
Turtleford (TUR)—3, 4, 7		
Watson Lake (WLK)—1, 5, 7		

**Table A2:** University of British Columbia horticulture greenhouse stock fertilizer solution.

Macronutrient	NH_4_	K	Na	Ca	Mg	NO_3_	Cl	S	HCO_3_	P	Si
mmol/L	0.1	5.9	0.4	6.4	3.2	16.3	2.3	3.3	0.1	1.42	0.05
**Micronutrient**	**Fe**	**Mn**	**Zn**	**B**	**Cu**	**Mo**					
µmol/L	39	1	2.7	39	0.9	1					

**Table A3:** Fracking wastewater composition used in the greenhouse study.

Element	Concentration (mg/L)
Calcium	1960
Magnesium	239
Sodium	21,000
Potassium	709
Iron	53.8
Zinc	<1
Arsenic	<0.04
Cadmium	<0.01
Chromium	<0.04
Copper	0.084
Lead	0.0619
Nickel	0.1001

**Table A4:** Mortality and average height of 32 poplar and willow genotypes from screening trial 1.

		Mortality (%)		Average Height (cm)		
Species	Genotype	30 mM NaCl	80 mM NaCl	0 mM NaCl	30 mM NaCl	80 mM NaCl
*Populus balsamifera*	BOY-6	100	100	77.2	N/A	N/A
	FTM-1	75	100	96.8	79.5	N/A
	FTM-2	75	100	94.6	77.1	N/A
	FTM-6	25	100	92.0	71.4	N/A
	FNO-1	100	100	121.2	N/A	N/A
	FNO-5	100	100	127.5	N/A	N/A
	FNO-7	25	100	118.3	105.0	N/A
	GPR-1	50	100	84.5	72.7	N/A
	GPR-10	100	100	98.2	N/A	N/A
	LOR-3	66	100	87.6	58.6	N/A
	LOR-5	50	100	79.2	70.8	N/A
	TUR-3	100	100	74.7	N/A	N/A
	TUR-4	50	100	82.7	56.6	N/A
	TUR-7	33	100	81.4	51.9	N/A
	WLK-1	100	100	89.2	N/A	N/A
	WLK-5	25	100	107.7	69.1	N/A
	WLK-7	100	100	98.0	N/A	N/A
*Salix eriocephala*	CAM-2	0	0	137.1	126.9	99.9
	CAM-5	0	0	92.3	101.7	71.3
	CAM-7	0	0	126.8	109.8	96.4
	DRU-2	0	0	107.7	125.6	107.2
	DRU-4	0	0	139.8	134.1	120.4
	DRU-6	0	0	156.4	123.0	73.0
	LAR-1	0	75	87.3	107.2	67.4
	LAR-3	0	0	137.0	132.8	126.3
	LAR-5	0	0	127.0	144.9	125.4
	PAL-1	0	0	100.6	102.5	82.2
	PAL-4	0	75	107.5	118.0	89.4
	STL-2	0	0	116.6	128.0	118.4
	STL-3	0	0	126.7	164.9	121.7
	STL-4	0	0	135.5	148.1	120.2
